# Chronic Liver Disease among Patients Admitted in the Department of Internal Medicine of a Tertiary Care Centre: A Descriptive Cross-sectional Study

**DOI:** 10.31729/jnma.8092

**Published:** 2023-03-31

**Authors:** Sujan Chandra Poudel, Abishkar Acharya, Sujata Maharjan, Saroj GC, Reshika Shrestha, Suraksha Thapa, Shekhar Poudel

**Affiliations:** 1 Department of Cardiology, Manmohan Cardiothoracic Vascular and Transplant Center, Maharajgunj, Kathmandu, Nepal; 2 Department of Internal Medicine, Kathmandu Medical College and Teaching Hospital, Sinamangal, Kathmandu, Nepal; 3 Bajrabarahi Chapagaun Hospital, Bajrabarahi, Lafitpur, Nepal; 4 Maharajgunj Medical Campus, Maharajgunj, Kathmandu, Nepal; 5 Nepalese Army Institute of Health Sciences, Sanobharyang, Kathmandu, Nepal; 6 Department of Gastroenterology, Kathmandu Medical College and Teaching Hospital, Sinamangal, Kathmandu, Nepal

**Keywords:** *alcoholic liver diseases*, *liver diseases*, *prevalence*

## Abstract

**Introduction:**

Chronic liver disease with cirrhosis is a significant cause of morbidity and mortality not only in developed but also in developing countries. Many patients already develop complications before hospital admission and require intensive medical care during hospital treatment. The main objective of the study was to find out the prevalence of the chronic liver disease among patients admitted in the Department of Internal Medicine of a tertiary care centre.

**Methods:**

A descriptive cross-sectional study was conducted among patients admitted to the Department of Internal Medicine of a tertiary care centre from 1 January 2022 to 31 March 2022. Ethical approval was obtained from the Ethical Review Board (Reference number: 2211202105). The patient admitted in the Department during the study period was included and those who do not gave consent were excluded. Convenience sampling method was used. Point estimate and 95% Confidence Interval were calculated.

**Results:**

Out of 447 patients, the prevalence of chronic liver disease was 93 (20.8%) (17.04-24.56, 95% Confidence Interval). The mean age of the patients was 49.69±10.94 years and among them, males were 64 (68.82%).

**Conclusions:**

The prevalence of the chronic liver disease among patients admitted to the Department of Internal Medicine of a tertiary care centre was lower than in other studies conducted in similar settings.

## INTRODUCTION

Chronic liver disease (CLD) is characterised by a continuous process of inflammation, destruction, and regeneration leading to fibrosis and cirrhosis with declining liver activities over a period of more than 6 months.^1^ Most cases are attributed to excessive alcohol consumption, viral hepatitis, or non-alcoholic fatty liver disease worldwide.^2^ The clinical course of the disease is usually fatal.^3^

Cirrhosis is currently the 11^th^ most common cause of death globally and liver cancer is the 16^th^ leading cause of death; combined, they account for 3.5% of all deaths worldwide.^4^ Chronic liver disease with cirrhosis is a significant cause of morbidity and mortality not only in developed but also in developing countries.^1^ Many patients already develop complications before hospital admission and require intensive medical care during hospital treatment.

This study aimed to find out the prevalence of the chronic liver disease among patients admitted in the Department of Internal Medicine of a tertiary care centre.

## METHODS

A descriptive cross-sectional study was conducted among patients admitted to the Department of Internal Medicine of the Kathmandu Medical College and Teaching Hospital, Sinamangal, Kathmandu, Nepal from 1 January 2022 to 31 March 2022. Ethical approval was obtained from the Ethical Review Board (Reference number: 2211202105). Patients admitted to the Department of Internal Medicine during the study period were included in the study. Those who do not give written consent were excluded from the study. A convenience sampling method was used. The sample size was calculated using the following formula:


$$\begin{array}{ll} \text{n} & ={\text{Z}}^{2}\times \frac{\text{p}\times \text{q}}{{\text{e}}^{\text{2}}}\\ & =1.96^{2}\times \frac{0.50\times 0.50}{0.05^{2}}\\ & =385 \end{array}$$

Where,

n = minimum required sample size
Z = 1.96 at 95% Confidence Interval (CI)
p = prevalence taken as 50% for maximum sample size calculation
q = 1-p
e = margin of error, 5%

Thus, the calculated minimum required sample size was 385. However, 447 patients were taken for the study.

Pre-determined proforma was used as the tool for data collection. All patients were subjected to detailed clinical and laboratory data. Various biochemical studies like alkaline phosphatase (ALP), aspartate aminotransferase (AST), alanine transaminase (ALT), total bilirubin, direct bilirubin, PT/INR, Serum albumin, urea, creatinine, sodium was done. An upper gastrointestinal (UGI) Endoscopy was done to rule out varices and other conditions and diagnostic findings were documented. Abdominal ultrasound was done for liver and spleen size, parenchymal echogenicity, portal vein diameter, and ascites. Child-Turcotte-Pugh (CTP) score and Model for End Stage Liver Disease (MELD)-Na scores were calculated for all the patients.^5^

Data were entered and analysed using Microsoft Excel 2016. Point estimate and 95% CI were calculated.

## RESULTS

Among 447 patients, the prevalence of chronic liver disease was 93 (20.8%) (17.04-24.56, 95% CI). The mean age of the patients with chronic liver disease was 49.69±10.94 years and among them, males were 64 (68.82%), followed by female 29 (31.18%) (Table 1).

**Table 1:** Clinical presentation of patients with chronic liver disease (n= 93).

Sign and symptoms	n (%)
Ascites	65 (69.89)
Jaundice	61 (65.59)
Fatigue	45 (48.38)
Hematemesis	41 (44.08)
Peripheral oedema	39 (41.93)
Melena	38 (40.86)
Anorexia	38 (40.86)
Abdominal pain	33 (35.48)
Vomiting	32 (34.41)
Altered sensorium	31 (33.33)
Pallor	29 (31.18)
Low urine output	20 (21.50)
Fever	17 (18.27)
Other bleeding manifestation	6 (6.45)
Spider naevi	6 (6.451)

**Figure 1:**
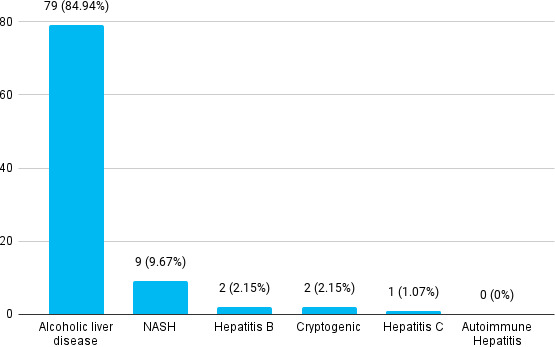
Underlying conditions of patients with chronic liver disease (n= 93).

Total 79 (84.94%) had alcoholic liver disease followed by non-alcoholic steatohepatitis (NASH) which was present among 9 (9.67%) patients (Figure 1).

Ascites was found to be the most common complication 65 (69.89%), followed by oesophageal varices 50 (53.76%) (Table 2).

**Table 2:** Complications of patients with chronic liver disease (n= 93).

Complications	n (%)
Ascites	65 (69.89)
Oesophageal varices	50 (53.76)
Gastrointestinal bleeding	40 (43.01)
Portal hypertensive gastropathy	38 (40.86)
Portal hypertension	34 (36.55)
Hepatic encephalopathy	30 (32.26)
Spontaneous bacterial peritonitis	8 (8.60)
Hepatorenal syndrome	7 (7.52)

**Figure 2:**
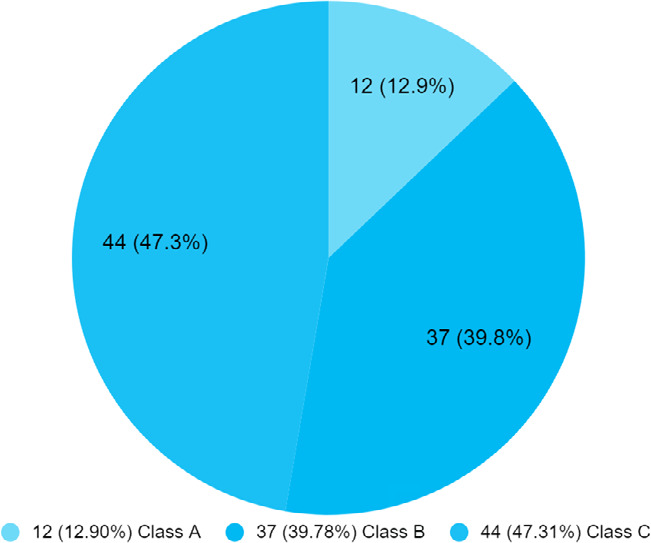
Child-Turcotte-Pugh score of patients with chronic liver disease (n= 93).

Death was recorded in 7 (7.53%) patient. The patient with Child C score was highest in 44 (47.31%) (Figure 2).

The mean MELD-Na score was 20.93±9.25. A total of 36 (38.71%) had MELD-Na score <17 (Table 3).

**Table 3:** MELD-Na Score of patients with chronic liver disease (n= 93).

Grading	n (%)
<17	36 (38.71)
17-20	13 (13.98)
21-22	2 (2.15)
23-26	18 (19.35)
27-31	9 (9.67)
≥32	15 (16.13)

## DISCUSSION

In our study, the prevalence of CLD was found to be 20.80%. The disease burden of CLD is increasing over the period of time.^6^ Studies showed the prevalence of CLD was 11.78% (1988-1994), 15.66% (1999-2004), and 14.78% (2005-2008).^2^ Similarly, in other studies, the prevalence was found to be 56.9% which is higher than our finding.^7^ Similarly in one of the studies, the prevalence of CLD is estimated to be 1.5 billion worldwide.^8^

The mean age of the patients with chronic liver disease was 49.69±10.94 years in our study. This is similar to the study, in which, the mean age of patients was 41.9±14.8 years,^7^ and in other similar studies the mean age was 45.8±10.45 years.^9^ In our study, among CLD patients, males (68.82%) were followed by females (31.18%). Similar to our study, one of the study showed the prevalence among males to be 70% and 30% were female,^7^ another similar study reported 88.3% males and 11.7% females.^9^

Cirrhotic patients present commonly with ascites, gastrointestinal bleeding, jaundice, low urine output and altered sensorium.^9^ In our study the most common signs and symptoms of patients with CLD was found to be ascites (69.89%) followed by jaundice (65.59%), which is similar to one of the studies, in which ascites was present in 74.3% of the patient followed by jaundice in 36.3%.^9^ Similar to our study gastrointestinal bleeding was (43.4%).^9^ In our study, the least presenting symptom and sign of CLD were found to be fever (18.27%) and spider naevi (6.47%) which is similar to this literature.^9^

Initially, viral hepatitis was supposed to be one of the leading causes of CLD but over the period of time, the main risk factor has been shifted to alcohol consumption.^8^ In one of the literature, it is found that, alcohol-related liver disease was responsible for 23.6 million cases of compensated cirrhosis, and 2.46 million cases of decompensated cirrhosis.^10^ In our study the most common underlying condition was found to be alcoholic liver disease (84.94%) which is similar to the study in which alcoholic liver disease was most common (75.7%).^7^ Non-alcoholic steatohepatitis (NASH) was present among 9.67% of patients. According to the study, the prevalence of non-alcoholic liver disease is increasing with time.^11^ The prevalence of non-alcoholic fatty liver disease (NAFLD) was 29.62%. This can be because of changing lifestyles and diets leading to obesity and NAFLD.^12^ We had no case of autoimmune hepatitis but in one of the studies, autoimmune hepatitis as one of the underlying conditions for CLD was found in 8.47%.^9^ In a study, the main underlying condition was alcohol (60.8%) which is similar to our study, which is followed by infections (14.9%). The least common was autoimmunity (1.4%), which is similar to our study.^7^

Major complications of cirrhosis include ascites, spontaneous bacterial peritonitis, hepatic encephalopathy, portal hypertension, variceal bleeding, and hepatorenal syndrome.^13^ In our study, ascites (69.89%) was the most common complication followed by oesophageal varices (53.76%) whereas, hepatorenal syndrome (7.52%) was the least common. This is similar to one of the studies, in which, the most common complication was ascites (78.6%), followed by variceal bleeding (43.4%). Similar to our study, HRS (2.7%) is relatively lower than other complications.^9^ In another similar study, the most common complication was spontaneous bacterial peritonitis (SBP) (20.2%), followed by hepatic encephalopathy (HE) (14.9%).^7^

CTP and MELD-Na scores are used to predict the prognosis of patients with liver disease.^5^ In our study, the patient with Class C CTP score was the highest in number (47.31%), followed by Class B (39.78%) and Class A (12.90%). Similar to our study, in one of the studies 50% had Class C CTP score, 40.4% had Child B and Child A was seen in only 9.8% of patients.^9^ In our study, the mean MELD-Na score was 20.93±9.25 which is similar to one of the studies.^9^ In our study the total mortality was (7.53%) of patients which is similar to the study (7.8%).^9^ The estimated death of CLD was 1-32 million in 2017 and the global burden due to CLD is increasing.^6^

The limitation of this study is since the study is conducted in a single centre, the results of the study cannot be generalised. Since it is a descriptive cross-sectional study, the association between cause and disease condition could not be established. Further analytical studies should be conducted to find out the correlation between exposure and outcome.

## CONCLUSIONS

The prevalence of chronic liver disease among patients admitted to the Department of Internal Medicine of a tertiary care centre was lower than in other studies conducted in similar settings. However, concerned stakeholders should emphasise targeted public health strategies, monitoring of at-risk populations about potential risk factors, and strengthening the healthcare system to decrease the burden of chronic liver disease.
